# Molecular and epigenetic profiles of BRCA1-like hormone-receptor-positive breast tumors identified with development and application of a copy-number-based classifier

**DOI:** 10.1186/s13058-018-1090-z

**Published:** 2019-01-25

**Authors:** Youdinghuan Chen, Yue Wang, Lucas A. Salas, Todd W. Miller, Kenneth Mark, Jonathan D. Marotti, Arminja N. Kettenbach, Chao Cheng, Brock C. Christensen

**Affiliations:** 1 Department of Epidemiology, Lebanon, USA; 2 Department of Molecular and Systems Biology, Lebanon, USA; 3 Department of Pathology and Laboratory Medicine, Lebanon, USA; 4 Department of Biochemistry and Cell Biology, Lebanon, USA; 5 Department of Biomedical Data Science, Lebanon, USA; 6 Department of Community and Family Medicine, Dartmouth-Hitchcock Medical Center, 660 Williamson, HB 7650. One Medical Center Drive, Lebanon, NH 03756 USA; 7 Present address: Department of Medicine, Baylor College of Medicine, Room ICTR 100D, One Baylor Plaza, Houston, TX 77030 USA

**Keywords:** BRCA1, BRCAness, Breast cancer, DNA methylation, Homologous recombination, Precision medicine

## Abstract

**Background:**

*BRCA1*-mutated cancers exhibit deficient homologous recombination (HR) DNA repair, resulting in extensive copy number alterations and genome instability. HR deficiency can also arise in tumors without a *BRCA1* mutation. Compared with other breast tumors, HR-deficient, BRCA1-like tumors exhibit worse prognosis but selective chemotherapeutic sensitivity. Presently, patients with triple negative breast cancer (TNBC) who do not respond to hormone endocrine-targeting therapy are given cytotoxic chemotherapy. However, more recent evidence showed a similar genomic profile between *BRCA1*-deficient TNBCs and hormone-receptor-positive tumors. Characterization of the somatic alterations of BRCA1-like hormone-receptor-positive breast tumors as a group, which is currently lacking, can potentially help develop biomarkers for identifying additional patients who might respond to chemotherapy.

**Methods:**

We retrained and validated a copy-number-based support vector machine (SVM) classifier to identify HR-deficient, BRCA1-like breast tumors. We applied this classifier to The Cancer Genome Atlas (TCGA) and Molecular Taxonomy of Breast Cancer International Consortium (METABRIC) breast tumors. We assessed mutational profiles and proliferative capacity by covariate-adjusted linear models and identified differentially methylated regions using *DMRcate* in BRCA1-like hormone-receptor-positive tumors.

**Results:**

Of the breast tumors in TCGA and METABRIC, 22% (651/2925) were BRCA1-like. Stratifying on hormone-receptor status, 13% (302/2405) receptor-positive and 69% (288/417) triple-negative tumors were BRCA1-like. Among the hormone-receptor-positive subgroup, BRCA1-like tumors showed significantly increased mutational burden and proliferative capacity (both *P* < 0.05). Genome-scale DNA methylation analysis of BRCA1-like tumors identified 202 differentially methylated gene regions, including hypermethylated *BRCA1*. Individually significant CpGs were enriched for enhancer regions (*P* < 0.05). The hypermethylated gene sets were enriched for DNA and chromatin conformation (all Bonferroni *P* < 0.05).

**Conclusions:**

To provide insights into alternative classification and potential therapeutic targeting strategies of BRCA1-like hormone-receptor-positive tumors we developed and applied a novel copy number classifier to identify BRCA1-like hormone-receptor-positive tumors and their characteristic somatic alteration profiles.

**Electronic supplementary material:**

The online version of this article (10.1186/s13058-018-1090-z) contains supplementary material, which is available to authorized users.

## Background

Germline mutation in the *BRCA1* gene is associated with an increased lifetime risk of breast cancer alongside earlier disease onset and predisposition to the more aggressive triple-negative disease subtype [1–4]. The enhanced risk and high penetrance of breast cancer due to a *BRCA1* germline mutation are attributable to the tumor-suppressor role of the BRCA1 protein, which modulates homologous recombination (HR)-dependent DNA repair [4–6]. *BRCA1*-related HR deficiency is associated with large-scale chromosomal breaks, extensive copy number alterations, and genome instability [7, 8]. However, HR deficiency is not limited to cancers carrying a *BRCA1* mutation. Epigenetic inactivation of *BRCA1*, as well as germline or somatic alteration of other HR-family genes, can serve as alternative mechanisms driving HR deficiency, resulting in a BRCA1-like phenotype also known as BRCAness [3, 4, 9–11]. Similar to *BRCA1*-mutated cancers, BRCA1-like cancers are aggressive and typically exhibit poor prognosis. However, BRCA1-like cancers are more sensitive to chemotherapy, evidenced in both experimental work and patient studies [4, 10–12]. Lacking HR DNA repair can selectively sensitize BRCA1-deficient cancer cells to DNA cross-linking, alkylating, and double stranded break-inducing agents as well as poly-ADP ribose polymerase (PARP) inhibitors [5, 6], and improved survival outcomes are observed after high-dose platinum-based chemotherapeutic and PARP inhibitor treatment in patients with BRCA1-like breast tumors [10, 11, 13]. The TALORx trial evaluating the potential benefit of chemo-endocrine versus endocrine therapy alone in patients with hormone-receptor-positive human epidermal growth factor 2 receptor (HER2)-negative cancer and intermediate OncotypeDX, recurrence scores showed mostly equivocal results [14]. However, some benefit of chemotherapy was observed in younger women with intermediate scores, potentially attributable to responses in patients with hormone-receptor-positive BRCA1-like tumors, which are diagnosed at a significantly younger age than non-BRCA1-like tumors.

BRCA1-like breast tumors harbor extensive, characteristic genomic alterations. Genomic analyses show the distinct molecular patterning of *BRCA1*-mutated, HR-deficient cancers compared to BRCA2-mutated amd HR-proficient cancers [15–17]. Another pronounced feature of BRCA1-like, HR-deficient cancers is the extensive copy number alterations. This molecular hallmark motivated the classification of HR-deficient tumors based on their copy number profiles. Initially, array comparative genomic hybridization (aCGH) copy number was used to characterize the BRCA1-like phenotype and led to the development of a tool to predict breast cancer in patients with a *BRCA1* mutation or promoter hypermethylation [10, 11, 18]. The aCGH copy-number features that distinguish BRCA1-like tumors led to the development of the BRCA1ness-MLPA assay, an experimental gold standard currently being tested in the clinical setting [19, 20]. More recently, the classification of HR deficiency has been adapted to measurement of copy number using higher-resolution approaches [11, 21].

A few studies have begun to characterize the molecular differences associated with BRCA1-related HR deficiency. HR-deficient cancers tend to exhibit more severe mutational burden and distinct mutational signatures [3, 15, 22]. Transcriptome-wide alterations have also been reported and used for defining HR-deficient gene signatures [12, 23, 24]. Further, HR deficiency is associated with global epigenetic changes and aberrant methylation of several HR family genes in cultured cells [25, 26]. However, these initial assessments of BRCA1-like molecular or cellular profiles often had limited sample sizes and varying results. Moreover, a description of biological differences between BRCA1-like and non-BRCA1-like tumors in large-scale cancer cohorts is currently lacking. Further, while prior work has shown the highly dysregulated epigenetic landscape in breast tumors compared to the normal breast, especially at early stages of cancer [2, 27], little is known about the epigenetic patterning of HR-deficient, BRCA1-like breast tumors relative to their non-BRCA1-like counterpart.

Here, we retrained and evaluated a classifier to identify BRCA1-like tumors using genome-wide copy number profiles, which can be measured by multiple platforms including genotyping array, methylation array, and next-generation sequencing [21]. We then applied this classifier to identify tumors exhibiting the HR-deficient, BRCA1-like phenotype in two large-scale breast cancer cohorts: The Cancer Genome Atlas (TCGA) [2] and the Molecular Taxonomy of Breast Cancer International Consortium (METABRIC) cohorts [28, 29]. In TCGA, for example, we detected nearly one third of breast tumors ofh the BRCA1-like phenotype, while only approximately 3% tumors had a *BRCA1* somatic alteration. Subsequently, we compared molecular, clinical, and epigenetic characteristics associated with the BRCA1-like phenotype, restricting our analyses to hormone-receptor-positive breast cancer (i.e. breast tumors expressing estrogen receptor (ER), progesterone receptor (PR), and/or human epidermal growth factor receptor 2 (HER2). We focused on these tumors because we anticipate their distinct molecular profile, which could render them responsive to the cytotoxic chemotherapy typically given to patients with triple negative breast cancer (TNBC) [30–32].

## Materials and methods

### Training, testing, and experimental validation of the BRCA1-like classifier

#### Data sets and samples

The training data set for developing a support vector machine (SVM) BRCA1-like classifier consists of a relatively balanced number of BRCA1-like and non-BRCA1-like breast tumors from the Netherlands Cancer Institute (Joosse data set, GSE9021 and GSE9114, *n* = 74 total) [16, 17]. The *BRCAx* data set, which consists of BRCA1/2-like and non-BRCA1/2-like breast tumors measured on the same aCGH copy number array platform of the Netherland Cancer Institute (GSE18626, *n* = 106) [33], was used as the independent validation set (Table 1). Breast cancer cell lines with genome-wide copy number data (available from the Cancer Cell Line Encyclopedia (CCLE) [34]) and BRCA1ness-MLPA assay profiles (seven collected in-house, three recently published [35]) were used as an additional validation set (Additional file 1: Table S1).

**Table 1:** Data sets used in this study. Number of samples (*n*) for a given data set indicates those with support vector machine (SVM)-predicted BRCA1-like status based on copy number data

Data set (*n*)	Percentage ER positivity^a^	Percentage Predicted BRCA1-like	Purpose	Data types analyzed	Accession (if applicable)	Refs.
Joosse et al. (74)	42.9 (27/63)	47.3 (35/74)	BRCA1-like classifier training	Copy number	GSE9021, GSE9114	[16, 17]
BRCAx (106)	70.7 (41/58)	19.8 (21/106)	BRCA1-like classifier validation	Copy number	GSE18626	[33]
CCLE breast cancer cell lines (10)	10.0 (1/10)	60.0 (6/10)	BRCA1-like classifier experimental validation	Copy number, MLPA	Additional file 1: Table S1; *portals.broadinstitute.org/ccle*	[34, 35]
TCGA breast cancer (957)	77.1 (704/913)	32.2 (308/957)	BRCA1-like differential analyses	Copy number, mutation, gene expression, DNA methylation, clinical	*synapse.org* *;* *gdc.cancer.gov*; *gdac.broadinstitute.org* *;*	[2, 39–41]
METABRIC (1968)	76.3 (1501/1968)	17.4 (343/1968)	BRCA1-like differential analyses	Copy number, gene expression, clinical	*cbioportal.org*	[28, 29]

*ER* estrogen receptor, *TCGA* The Cancer Genome Atlas, *CCLE* Cancer Cell Line Encyclopedia, *METABRIC* Molecular Taxonomy of Breast Cancer International Consortium

^a^ER status is not reported, is unknown, or is equivocal in a subset of Joosse et al., BRCAx and TCGA breast tumors. Such tumors were excluded from percentage ER positivity calculation

#### Classifier training and testing

**Fig. 1:**
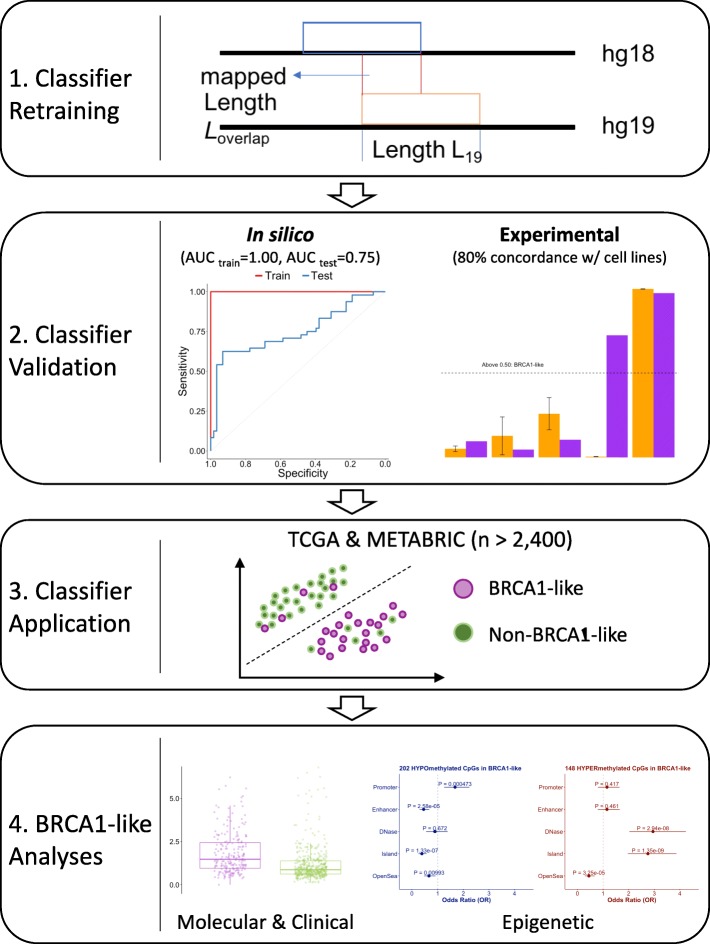
Workflow for developing a support vector machine (SVM) BRCA1-like classifier and application to publicly available datasets for biological discovery. In step 1, a new SVM-based BRCA1-like classifier is trained on re-processed and normalized array copy number data. In step 2, the receiver-operation characteristic (ROC) curves were used for evaluating our BRCA1-like classifier in a training and test set (AUC _training_ = 1.00, AUC _test_ = 0.75). In step 3, we applied the SVM classifier to tumors in the The Cancer Genome Atlas (TCGA) and Molecular Taxonomy of Breast Cancer International Consortium (METABRIC) data sets. Finally, in step 4, we performed bioinformatics and statistical analyses attempting to understand the biological characteristics of hormone-receptor-positive breast tumors predicted to be BRCA1-like by our SVM classifier

Figure 1 summarizes the workflow for development and application of the BRCA1-like classifier in this study. Briefly, the training set hg18 copy number features previously known to distinguish BRCA1-like and non-BRCA1-like breast tumors [10, 16] were mapped onto the hg19 reference assembly. Genomic annotation files used for lift-over were downloaded from the UCSC Genome Browser (*hgdownload.cse.ucsc.edu*). We then used an in-house algorithm to map and normalize segmented copy numbers as shown in Additional file 2: Figure S1.

Next, normalized gene copy number profiles were used to retrain a support vector machine (SVM) BRCA1-like classifier in a similar manner as in published reports [11, 21]. Briefly, our BRCA1-like classifier solves the following optimization problem:

$$ \underset{\boldsymbol{\theta}, {\boldsymbol{\theta}}_{\mathbf{0}}}{\min}\frac{1}{2}{\boldsymbol{\theta}}_{\boldsymbol{i}}^T{\boldsymbol{\theta}}_{\boldsymbol{i}}+C\sum \limits_{i=1}^n{\zeta}_i $$

where *θ*_*i*_*, θ*_*0*_ are model weights and regression intercept for the *i*-th tumor, respectively. *C* (= 8.00) and *ζ*_*i*_ are SVM hyperparameters. We applied sigmoidal kernel smoothing and ten-fold cross validation. The probability of the *i-*^*th*^ sample belonging to the BRCA1-like class was calculated as:

$$ \Pr \left({y}_i=1\ |\ {\boldsymbol{x}}_{\boldsymbol{i}},{\boldsymbol{\theta}}_{\boldsymbol{i}}\right)=\frac{1}{1+\exp \left(-{\boldsymbol{\theta}}_0+{\boldsymbol{x}}_i^T{\boldsymbol{\theta}}_i\right)} $$

where ***x***_***i***_ is a vector of normalized copy number features and *y*_*i*_ is the binary BRCA1-like class (*y*_*i*_ = 1 indicates BRCA1-like). Above the probabilistic threshold of 0.50 (i.e. *Pr(y*_*i*_ *= 1) > Pr(y*_*i*_ *= 0)*), a tumor is defined as being BRCA1-like. The SVM classifier training and performance evaluation were implemented in the *e1071* and *pROC* R package, respectively [36–38].

#### Experimental validation of the retrained BRCA1-like classifier

To further validate the SVM classifier, the BRCA1ness-MLPA assay (MRC-Holland catalog number P376), a BRCA1-like experimental gold standard, was used [19]. To this end, the SVM BRCA1-like classifier was first applied to published breast cancer cell line copy number data measured by Affymetrix SNP6.0 array (available from *portals.broadinstitute.org/ccle*) [34]. Next, the BRCA1ness-MLPA assay was performed on breast cancer cell lines BT549, CAL51, CAL120, HCC1806, HDQ-P1, MDA-MB-231 and MDA-MB-468 obtained from American Type Culture Collection (ATCC), thawed and cultured < 4 months prior to assay. Authenticated by ATCC, the cell lines used are assumed to be mycoplasma-free. No further mycoplasma testing or authentication was performed after thawing: 200 ng DNA was precipitated using 0.5 μL of 20 mg/mL glycogen, 15 μL of 3 M sodium acetate, and 495 μL pure ethanol at − 20 °C for 3 h, followed by precipitation at 14,000*g for 30 min at 4 °C and a 70% ethanol rinse. Precipitated DNA was re-suspended at a final concentration of 25 ng/μL and solubilized by incubating at 37 °C for 1 h. DNA from three fresh-frozen normal breast DNA (source: National Disease Research Interchange) and RPE1 cell line were used as copy number-neutral controls. The subsequent steps were based on the BRCA1ness-MLPA kit instructions (MRC-Holland, the Netherlands): 100 ng DNA was mixed with S4 Stabilizer and denatured at 98 °C for 5 min. Probe-DNA hybridization was performed at 60 °C for ≥ 16 h, followed by a 15-min ligation at 54 °C and 5-min ligase inactivation at 98 °C. Then, 35 cycles of PCR were performed, each with 30-s denaturation at 95 °C, 30-s annealing at 60 °C, and 60-s extension at 72 °C, followed by a 20-min final extension at 72 °C. Fragment analysis was performed by capillary electrophoresis in technical triplicate on the ABI3730 DNA Analyzer (Thermo Fisher Scientific, Waltham, MA, USA). The *Coffalyser.Net* software with default peak detection setting was used. BRCA1-like classification was executed by the nearest shrunken centroid method implemented in the *pamr* R package (v.1.55), and a probabilistic threshold of 0.50 recommended by the manufacturer was used to call BRCA1-like tumors. MLPA profiles for three additional cell lines (HCC70, MCF7, and MDA-MB-436) were recently published by Roig et al. [35]. SVM BRCA1-like status and MLPA profiles of the breast cancer cell lines used are listed in Additional file 1: Table S1.

#### Correlation of SVM BRCA1-like probability scores with published HR deficiency metrics

The Homologous Recombination Deficiency-Loss of Heterozygosity (HRD-LOH) [8, 13] and Large Scale Transition (LST) [7] scores for 717 TCGA breast tumors were downloaded from Marquard et al. [39]. The correlation between SVM BRCA1-like probability scores and HRD-LOH scores or LST scores was assessed by linear regression.

### BRCA1-like analyses

#### Data sets and samples

For the remainder of the study, we compared BRCA1-like and non-BRCA1-like receptor-positive breast tumors in two large-scale breast cancer cohorts: TCGA and METABRIC. SVM BRCA1-like status was estimated in the same manner as mentioned.

The TCGA breast cancer molecular and clinical data were downloaded from FireBrowse (*gdac.broadinstitute.org*), cBioPortal (*cbioportal.org*), SynapseTCGAlive (*synapse.org*), and the Genomic Data Commons (*gdc.cancer.gov*). We excluded 100 tumors for which TNBC status could not be ascertained and 18 additional *BRCA1/2*-mutated or *BRCA1*-hypermethylated tumors predicted to be non-BRCA1-like, leaving a total of 837 tumors (Additional file 1: Table S2A). DNA methylation and gene expression, measured by Illumina 450 K and RNA-seqV2/miRseq, respectively, were available for a subset of TCGA tumors.

METABRIC breast cancer clinical data, copy number, gene expression, and *BRCA1/2* somatic mutation profiles were downloaded from cBioPortal (*cbioportal.org*). DNA methylation and mutational burden/signature were not available for this data set. Among breast tumors with SVM BRCA1-like status, a large proportion (519/1985) with missing tumor stage and 12 classified as stage 0 (ductal carcinoma in situ) were excluded. Additionally, 37 non-BRCA1-like tumors with a *BRCA1/2* mutation were considered misclassified and were excluded, leaving 1429 tumors (Additional file 1: Table S2B).

#### Relation of genomic burden with BRCA1-like status

Mutational rates per mega base-pairs (Mb) were published by Kandoth et al. [40] and are available for 662 TCGA tumors. Given the large number of outliers and substantial variability in mutation rates, the association between mutation rates per Mb and SVM BRCA1-like status was assessed by a linear model with robust variance adjusting for subject age, tumor stage, and ER, PR, and HER2 positivity, implemented in R packages *MASS* (v.7.3.49), *sandwich* (2.4.0), and *lmtest* (v.0.9.35) with *type = HC0*. Somatic Mutational Signature 3, which is related to HR deficiency, and Mutational Signature 1, which is related to global CpG methylation, were published by Rosenthal et al. [41] and are available for 745 TCGA breast tumors. A linear model adjusting for the same potential confounders was used for comparison.

#### Survival analysis

A Cox proportional hazards regression model was used to test the relationship between overall survival and BRCA1-like status. For this analysis, we combined TCGA and METABRIC data sets and restricted our analysis to ER-positive or PR-positive and HER2-negative patients as follows:

$$ {\lambda}_i(t)={\lambda}_0(t)\ \exp \left({\beta}_{i, SV{M}_{BRCA1}}\mathrm{SVM}\_\mathrm{BRCA}{1}_{\mathrm{i}}+{\beta}_{i, Age}\ \mathrm{Ag}{\mathrm{e}}_{\mathrm{i}}+{\beta}_{i, Stage}\mathrm{Stag}{\mathrm{e}}_{\mathrm{i}}\right) $$

where *λ*_*i*_ and *λ*_*0*_ are hazard for *i*-th patient and baseline hazard assumed to be constant, respectively. *t* represents overall survival time in months, *SVM_BRCA1* represents the BRCA1-like status, *Age* represents age at diagnosis in years, and *Stage* is a binary variable representing early stage (II or lower) or late stage (III or higher). Administrative censoring was imposed at 5 years (60 months) of follow up. Cox regression and data visualization were implemented in R packages *survival* (v.2.41.3) and *survminer* (v.0.4.2).

#### DNA methylation processing and analysis

In the TCGA breast cancer data set, level 1 methylation intensity data files from the Illumina 450 K array were preprocessed by the *minfi* R/Bioconductor package (v.1.20). Based on the overall methylated-to-unmethylated intensity ratio, four samples classified as poor-performing outliers were excluded. CpG probes with *P* > 0.05 for detection in more than 20% samples were excluded from downstream analysis. The quality control procedure left 464,028 high-quality CpG probes in the final data set. *BRCA1* promoter hypermethylation was determined using four array CpGs (cg19531713, cg19088651, cg08993267, and cg04658354) as previously described; samples with mean beta values ≥ 0.20 were defined as *BRCA1* promoter-hypermethylated [42, 43].

To identify differentially methylated CpGs and gene regions in BRCA1-like tumors relative to non-BRCA1-like tumors predicted by the SVM classifier (*n* = 322 with no missing covariates), we applied the *DMRcate* algorithm (v.1.14.10) to 464,028 CpGs adjusting for subject age, tumor stage, and ER, PR, and HER2 status [44]. *DMRcate* first identified individually significant CpGs at a false discovery rate (FDR) < 0.05. A differentially methylated region (DMR) was then called if a given gene region had ≥ 10 significant CpGs within a 1-kb bandwidth and a Benjamini-Hochberg FDR < 0.05. Autosomal and chromosome X DMRs were identified separately to avoid bias driven by imprinting. Individually significant CpGs were used for genomic context enrichment against the 464,028-CpG universe set using the two-tailed Fisher’s exact test. CpGs with |%∆beta| ≥ 12.5% were used for unsupervised hierarchical clustering with Euclidean distance and complete linkage. DMR-associated genes were used as input for Gene Ontology: Biological Processes against the whole genome via the WebGestalt tool (*webgestalt.org*) [45]. The minimum and maximum number of genes required per pathway were 5 and 2000, respectively (default). Raw *P* values were adjusted by the Bonferroni method.

DNA methylation age (“epigenetic clock”) was inferred by applying the Horvath algorithm [46] to 450 K methylation beta-values in 322 breast tumors tested for differential methylation. To compare methylation age or chronological age between BRCA1-like and non-BRCA1-like tumors, separate linear models were built adjusting for tumor stage, ER, PR, and HER2 positivity.

#### Relation of candidate gene expression with BRCA1-like status

Linear models were used to compare Ki-67 (*MKI67*), *DNMT1/3A/3B*, and miR124–2 gene expression between BRCA1-like and non-BRCA1-like receptor-positive tumors adjusting for age, tumor stage, and ER, PR, and HER2 positivity.

## Results

### Development of a copy-number-based BRCA1-like classifier

Prior studies demonstrated the utility of array comparative genomic hybridization (aCGH) copy number profiles for BRCA1-like classification in breast tumors [16, 17, 21, 33]. To enable identification of BRCA1-like tumors measured by a non-aCGH copy number platform or analyzed by a more up-to-date genome assembly, such as The Cancer Genome Atlas (TCGA) data set, we first mapped copy number features previously used for BRCA1-like classification to the human hg19 reference genome followed by data re-normalization using an in-house pipeline (Additional file 2: Figure S1A). We then retrained a BRCA1-like classifier using support vector machine (SVM), a robust supervised-learning method that seeks a class-separating hyperplane within higher dimensional data [36]. Our SVM BRCA1-like classifier achieved acceptable performance in the training and the independent test set (AUC _training_ = 1.00, AUC _test_ = 0.75, Additional file 2: Figure S1B). In the Cancer Cell Line Encyclopedia (CCLE) data set with publicly available copy number data, SVM BRCA1-like status in breast cancer cell lines is 80% (8/10) concordant with the BRCA1ness-MLPA assay profile measured in house (Additional file 1: Table S1). When applied to TCGA breast tumors, SVM BRCA1-like probability scores were highly correlated with existing HR deficiency metrics [39] (both *P* < 2.2E-16; Additional file 2: Figure S1C-D). Figure 1 summarizes the workflow for the present study.

### Molecular and clinical characteristics related with the BRCA1-like phenotype

**Fig. 2:**
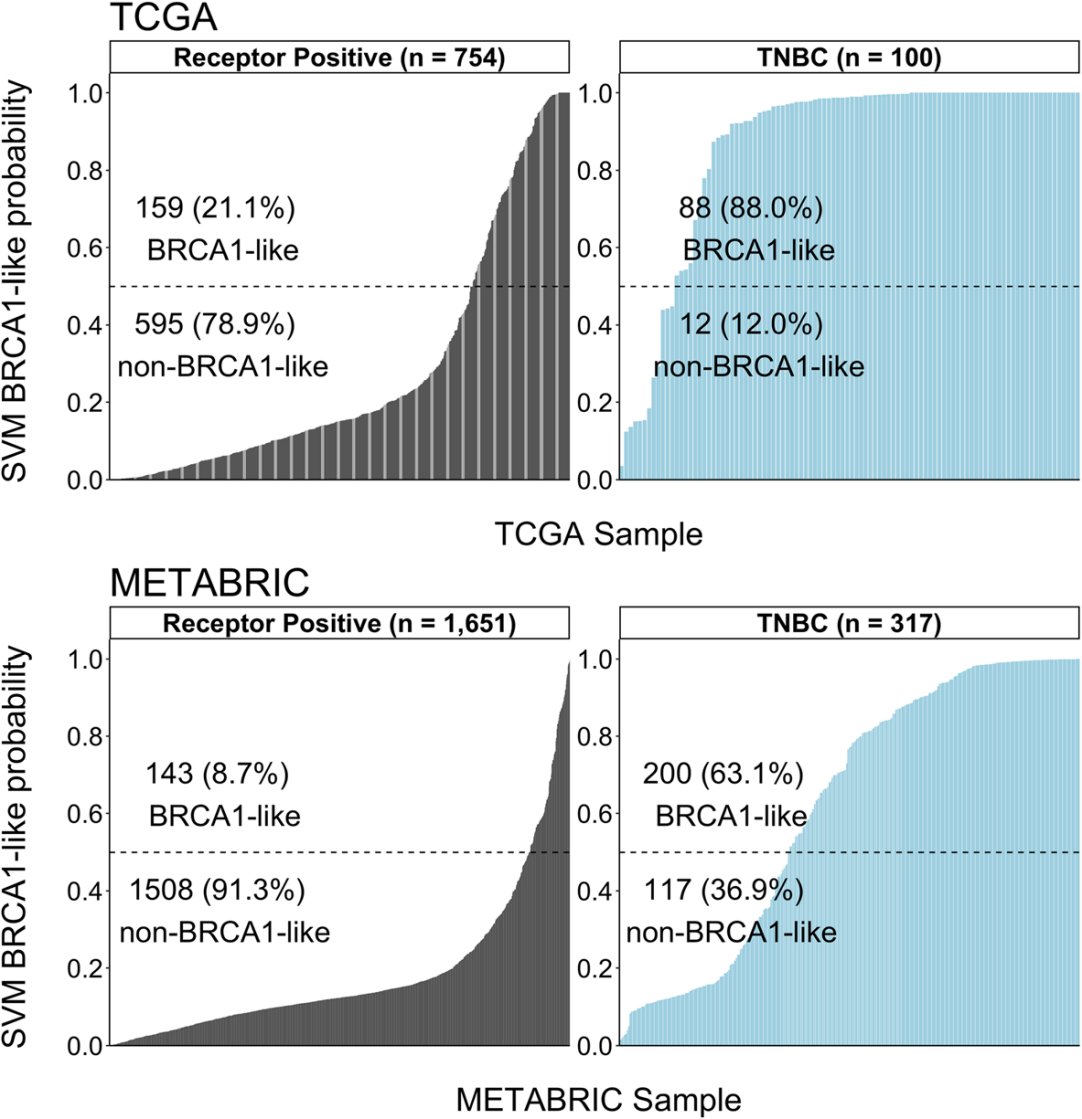
Distribution of BRCA1-like probability scores in The Cancer Genome Atlas (TCGA) (top panel) and Molecular Taxonomy of Breast Cancer International Consortium (METABRIC) (bottom panel) data sets, stratified by hormone-receptor-based subtypes. Each vertical bar represents a patient. The height of the bar represents the probability score of being BRCA1-like assigned by our support vector machine (SVM) copy number classifier. TNBC, triple negative breast cancer

As expected, a large proportion (69%, 288/417) of all triple negative breast cancer (TNBC) was predicted to be BRCA1-like [47, 48] (Table 2, Additional file 1: Table S3 and Fig. 2). Among all BRCA1-like tumors, 36% (237/651) were positive for estrogen receptor (ER) and 14% (93/651) for human epidermal growth factor 2 (HER2). In addition, in the TCGA data set where race information is available, 21.3% (60/282) of African American subjects were classified as having BRCA1-like tumors compared to 10.4% (61/585) classified as having non-BRCA1-like tumors (Additional file 1: Table S3A). In other words, there is a 2.32-fold (95% CI = 1.54–3.49, *P* = 2.59E-5) increase in the proportion of African American subjects classified as having BRCA1-like compared to non-BRCA1-like tumors, and the increased prevalence of TNBC in African Americans is established [22, 49] (Additional file 1: Table S3A). We also noted the difference in BRCA1-like probability score distribution between TCGA and METABRIC. This difference could be explained by the differential subject characteristics including younger age at diagnosis and higher cancer stage in the TCGA than the METABRIC data set (linear regression *P* = 2.62-E7 and Fisher’s exact test *P* < 2.2E-16, respectively).

**Table 2:** Prevalence of BRCA1-like phenotype in the large-scale breast cancer cohorts, TCGA and METABRIC

	TCGA			METABRIC		
	BRCA1-like	non-BRCA1-like	*P* value ^§^	BRCA1-like	non-BRCA1-like	*P* value ^§^
n	308	649		343	1625	
Age, years (mean (sd))	56.57 (13.26)	59.33 (13.06)	0.002	56.74 (13.93)	62.02 (12.56)	< 0.001
Stage (%)			0.22			0.35
Stage I-II	238 (77.3)	473 (72.9)		220 (64.1)	1106 (68.1)	
Stage III-IV	63 (20.5)	165 (25.4)		26 (7.6)	102 (6.3)	
(Missing)	7 (2.3)	11 (1.7)		97 (28.3)	417 (25.7)	
ER (%)			< 0.001			< 0.001
Positive	129 (41.9)	575 (88.6)		108 (31.5)	1393 (85.7)	
Negative	166 (53.9)	43 (6.6)		235 (68.5)	232 (14.3)	
(Missing)	13 (4.2)	31 (4.8)		0 (0.0)	0 (0.0)	
PR (%)			< 0.001			< 0.001
Positive	97 (31.5)	514 (79.2)		57 (16.6)	980 (60.3)	
Negative	195 (63.3)	104 (16.0)		286 (83.4)	645 (39.7)	
NA	16 (5.2)	31 (4.8)		0 (0.0)	0 (0.0)	
HER2 (%)			0.59			0.98
Positive	50 (16.2)	93 (14.3)		43 (12.5)	200 (12.3)	
Negative	161 (52.3)	333 (51.3)		300 (87.5)	1425 (87.7)	
(Missing)	97 (31.5)	223 (34.4)		0 (0.0)	0 (0.0)	
Any ER, PR, or HER2 positivity (%)			< 0.001			< 0.001
Yes	159 (51.6)	595 (91.7)		143 (41.7)	1508 (92.8)	
No	88 (28.6)	12 (1.8)		200 (58.3)	117 (7.2)	
Cannot be determined	61 (19.8)	42 (6.5)		0 (0.0)	0 (0.0)	

*TCGA* The Cancer Genome Atlas, *METABRIC* Molecular Taxonomy of Breast Cancer International Consortium, *ER* estrogen receptor, *PR* progesterone receptor, *HER2* human epidermal growth factor receptor 2

^§^For any continuous measure (i.e. age), the two-tailed *t*-test was used. For any categorical measure, the two-tailed Fisher’s exact test was used

**Fig. 3:**
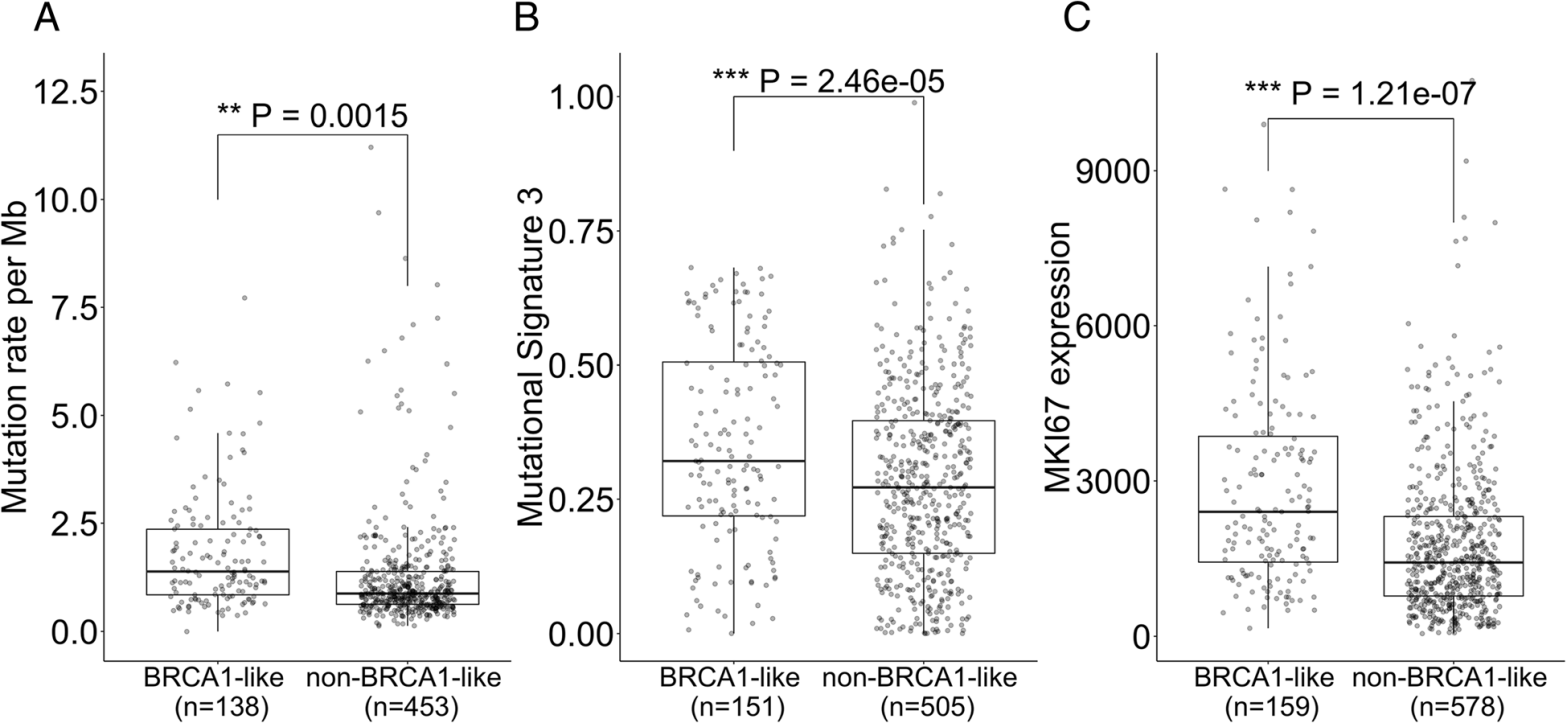
Molecular characteristics of support vector machine (SVM) BRCA1-like hormone-receptor-positive tumors in The Cancer Genome Atlas (TCGA). Comparison of whole exome sequencing-based mutation rates per mega base-pair (Mb) (**a**) and Somatic Mutational Signature 3 related with HR-deficiency (**b**), and Ki-67 (*MKI67*) gene expression as a surrogate marker for cellular proliferation (**c**). Variations in the number of tumors were due to data availability (see “Materials and methods”). *P < 0.05, **P < 0.01, ***P < 0.001

We detected 13% (302/2405) hormone-receptor-positive patients with breast cancer to have BRCA1-like tumors. Recent studies suggest that hormone-receptor-positive tumors could also exhibit HR deficiency and potentially benefit from chemotherapeutic treatments [30–32]. Hereafter, we restricted our analyses to hormone-receptor-positive breast tumors (Table 2 and Additional file 1: Table S3). In TCGA, BRCA1-like tumors exhibit greater mutational burden than their non-BRCA1-like counterpart (*P* < 0.01, Fig. 3a). Somatic Mutational Signature 3, inferred from exome sequencing and strongly related to HR deficiency [3, 15, 22, 50], was significantly elevated in BRCA1-like tumors (*P* < 0.001, Fig. 3b). These tumors also demonstrated enhanced proliferative capacity, indicated by increased Ki-67 gene expression (*P* < 0.001, Fig. 3c and Additional file 2: Figure S2). In addition, BRCA1-like status appeared to have a harmful association with 5-year overall survival based on Kaplan-Meier analysis in ER/PR-positive, HER2-negative tumors, and although the hazards ratio estimate indicated poorer prognosis in models adjusted for potential confounders, the results were not statistically significant (Additional file 2: Figure S3).

### Epigenetic characteristics of hormone-receptor-positive BRCA1-like breast tumors

Promoter hypermethylation of *BRCA1* or other HR-family genes (e.g. *RAD51C*) is a known mechanism driving HR deficiency and BRCAness [2–4, 15, 22, 31, 42]. Inactivation of *BRCA1* by promoter hypermethylation also shows higher prevalence in the triple-negative subtype [2, 4, 48]. However, the relationships between the genome-scale DNA methylation pattern and the HR-deficient phenotype in hormone-receptor-positive breast cancer has not been previously reported. We applied the *DMRcate* algorithm to identify individual cytosine-phosphate-guanine (CpG) sites and genomic regions harboring differential methylation in BRCA1-like relative to non-BRCA1-like tumors identified by our SVM BRCA1-like classifier [44]. This approach identified 350 CpGs with a FDR < 0.05 and |% ∆beta| ≥ 12.5%, which we define as differentially methylated. Unsupervised hierarchical clustering of these differentially methylated loci separated tumors into two major clusters. Methylation of CpGs in the “BRCA1-like Cluster” exhibited greater heterogeneity compared to CpGs in the “non-BRCA1-like cluster” (Additional file 2: Figure S5; mean inter-sample variances in methylation beta-values for BRCA1-like cluster are 0.0527 and 0.0371, respectively).

**Fig. 4:**
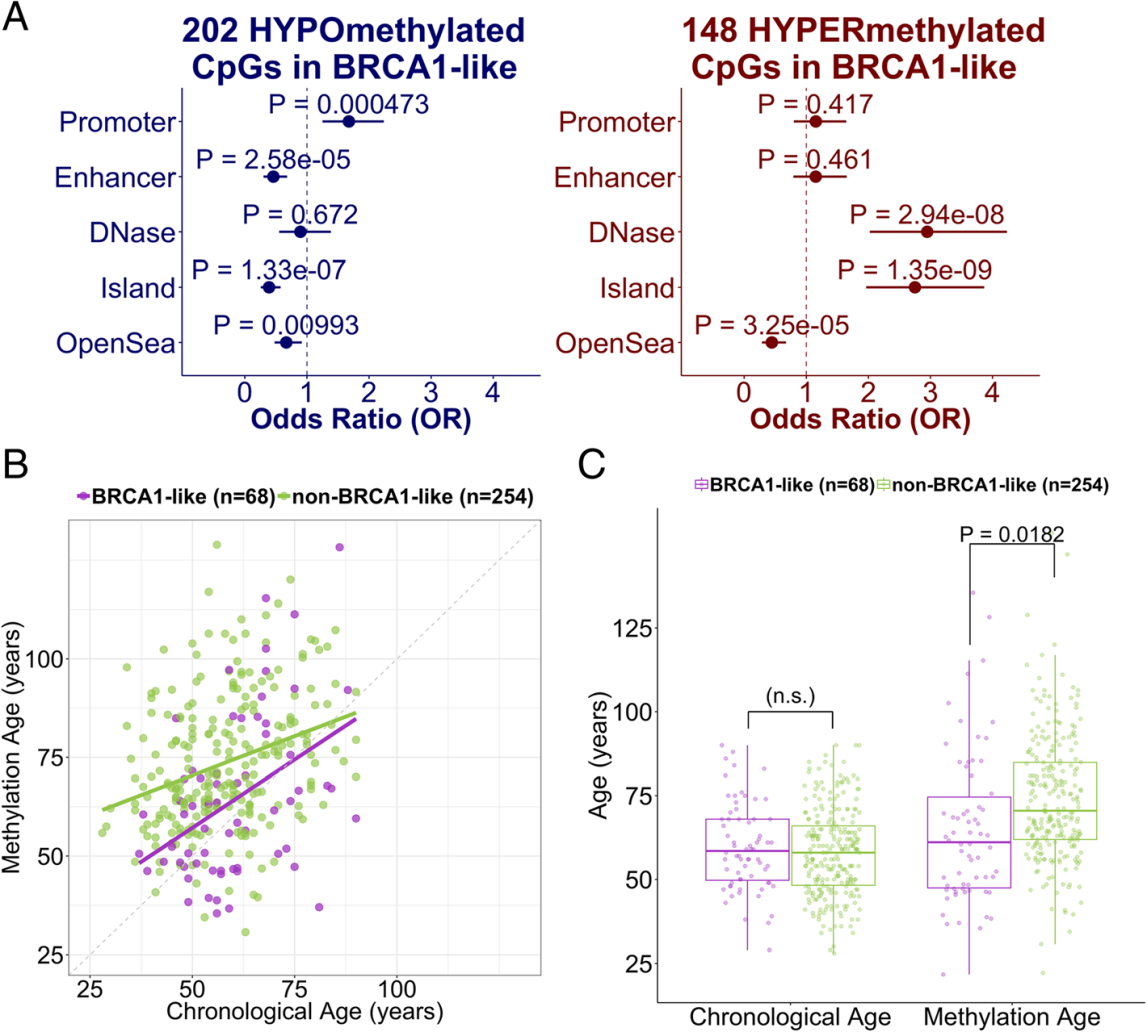
Differential DNA methylation in groups with BRCA1-like tumors relative to groups with non-BRCA1-like tumors defined by the support vector machine (SVM) BRCA1-like classifier among hormone-receptor-positive tumors in The Cancer Genome Atlas (TCGA). **a** Genomic context enrichment analysis of hypermethylated and hypomethylated CpGs. Solid dots and horizontal segments indicate odds ratios and 95% confidence intervals. *P* values were from the two-tailed Fisher’s exact test. **b** Correlation of the Horvath methylation age (“epigenetic clock”) with patient chronological age, colored by BRCA1-like status. **c** Comparison of chronological age or the Horvath methylation age between BRCA1-like and non-BRCA1-like hormone-receptor-positive tumors. *P* value indicates statistical significance from the covariate-adjusted linear model. n.s., not significant

We next investigated the biological relevance of the differentially methylated CpG loci and gene sets. Hypermethylated CpGs associated with SVM BRCA1-like status determined from the SVM classifier were enriched for CpG islands but not for promoter regions. Stratified by direction of the change in methylation, 202 of 350 hypomethylated CpGs overrepresented gene promoters (OR = 1.68, 95% CI = 1.25–2.24) and underrepresented enhancers (OR = 0.46, 95% CI = 0.30–0.68). There were 48 out of 350 differentially hypermethylated CpGs that were enriched for DNase I hypersensitivity sites associated with active chromatin and gene transcription (OR = 2.95, 95% CI = 2.02–4.23). Intriguingly, the hypermethylated and hypomethylated CpGs overrepresented and underrepresented the “CpG Island” genomic context, respectively (OR _Hyper_ = 2.75, 95% CI _Hyper_ = 1.96–3.87; OR _Hypo_ = 0.39, 95% CI _Hypo_ = 0.26–0.57). Both sets significantly underrepresented the “Open Sea” genomic context that has low CpG density (OR _Hyper_ = 0.45, 95% CI _Hyper_ = 0.29–0.67; OR _Hypo_ = 0.67, 95% CI _Hypo_ = 0.48–0.92) (Fig. 4a).

There were 108 and 94 gene regions that were hypermethylated and hypomethylated in BRCA1-like tumors, respectively (all with FDR < 0.05 and a minimum of 10 CpGs per kb; Additional file 1: Table S5A-B). The *BRCA1* locus had increased methylation in BRCA1-like tumors. In line with this key observation, *FANCF*, another member of the BRCA1/Fanconi anemia pathway [51], was also hypermethylated (Additional file 1: Table S5A). Hypermethylation of developmentally related genes, including *HOXB13*, *HOXD3*/*12*, and *FOXR1,* suggested that developmental signaling might be dysregulated in BRCA1-like tumors (Additional file 1: Table S5A). Many histone genes were also hypermethylated, hinting at potentially elevated DNA damage and genome instability in BRCA1-like tumors. miR124–2, a microRNA that negatively regulates cellular proliferation in breast cancer [52], showed hypermethylation and reduced expression in BRCA1-like tumors (*P* < 0.05; Additional file 2: Figure S4). Among the hypomethylated genes, E3 ubiquitin ligases (*HUWE1, UNKL*, and *VHL*) that also play a role in cell-cycle regulation [53] showed reduced methylation in BRCA1-like receptor-positive tumors (Additional file 1: Table S5B). Applying Gene Ontology: Biological Processes to 158 genes associated with the hypermethylated DMRs, we identified DNA conformation and chromatin assembly-related gene sets to be most hypermethylated (all with FDR < 0.05; Table 3). Gene sets related to developmental signaling and the cell cycle had mild enrichment (Additional file 1: Table S6A).

**Table 3:** Gene Ontology: Biological Processes for genes associated with hypermethylated gene regions

Accession	Description	Number of genes	Number of input genes	Expected ratio	Observed ratio	*P* value	Bonferroni *P* value
GO:0071103	DNA conformation change	248	10	1.39	7.20	1.24E-06	0.011
GO:0006323	DNA packaging	165	8	0.92	8.66	3.95E-06	0.017
GO:0031497	Chromatin assembly	128	7	0.72	9.77	7.44E-06	0.021
GO:0065004	Protein-DNA complex assembly	197	8	1.10	7.26	1.45E-05	0.031
GO:0006333	Chromatin assembly or disassembly	153	7	0.86	8.17	2.38E-05	0.041
GO:0006334	Nucleosome assembly	111	6	0.62	9.66	3.68E-05	0.045
GO:0071824	Protein-DNA complex subunit organization	224	8	1.25	6.38	3.65E-05	0.045

The complete list of pathways can be found in Additional file 1: Table S6A

Given the hypermethylation of many developmentally related genes and detection of developmental gene sets, we compared the DNA methylation age (“epigenetic clock”), a metric related to aging, cell-culture passage, and differentiation potential [46], between BRCA1-like and non-BRCA1-like tumors. Adjusting for tumor stage and hormone-receptor positivity, DNA methylation age was significantly lower in patients with BRCA1-like tumors (median 60.7 years, compared to non-BRCA1-like median 70.5 years; *P* < 0.05; Fig. 4b-c).

To confirm a distinct global DNA methylation landscape in BRCA1-like tumors, we compared gene expression of the *de novo* methyltransferases *DNMT3A/3B* and the maintenance methyltransferase *DNMT1* [54] between BRCA1-like and non-BRCA1-like tumors. All three methyltransferases were overexpressed in BRCA1-like tumors (all *P* < 0.001; Additional file 2: Figure S6). Likewise, Mutational Signature 1, contributed to by genome-wide cytosine-to-thymine deamination that acts on unmethylated Np*C*pG sequences [50], was significantly lower in BRCA1-like tumors (*P* < 0.01; Additional file 2: Figure S7).

## Discussion

In this study, we retrained a BRCA1-like classifier using genome-wide copy number and validated the classifier by *in silico* and experimental approaches. We estimated that 22% of all TCGA and METABRIC breast tumors were BRCA1-like, consistent with existing literature [4, 15]. Notably, 13% hormone-receptor-positive tumors were BRCA1-like. Therapeutic strategies such as cytotoxic chemotherapy more commonly used in the triple-negative disease setting might be an effective alternative for treating these tumors.

Among hormone-receptor-positive breast tumors, the BRCA1-like phenotype is associated with increased mutational burden, as demonstrated by elevated mutation rates. Expression of Ki-67, a surrogate marker for cellular proliferation, was increased in BRCA1-like receptor-positive tumors. These molecular hallmarks serve as evidence supporting the more aggressive character of BRCA1-like tumors.

The genome-scale DNA methylation profile of BRCA1-like tumors, identified by our SVM classifier, appeared distinct. Furthermore, we detected hypermethylation of gene sets related to chromatin and nucleosome assembly. Of note, the *BRCA1* locus showed increased DNA methylation in BRCA1-like tumors, supporting the existing concept that hypermethylation of HR-family genes could serve as a driver for HR deficiency. Detecting hypermethylation and reduced gene expression of miR124–2, a negative regulator of cell proliferation [52], is consistent with our Ki-67 gene expression analysis and further supports BRCA1-like tumors having a more aggressive phenotype.

Subsequently, differential methylation analysis comparing SVM-predicted BRCA1-like and non-BRCA1-like tumors identified 202 hypomethylated and 148 hypermethylated CpGs. Unsupervised hierarchical clustering of all 350 CpGs revealed a distinct “BRCA1-like cluster”, implying the potential utility of genome-scale DNA methylation as another biomarker to identify HR-deficient cancers, possibly in breast cancer biopsies shown to have similar methylation profiles to larger surgical blocks [55]. We also noticed the increased heterogeneity in this cluster relative to the “non-BRCA1-like cluster”, a hallmark of aggressive cancers [56]. In addition, this finding parallels the prior observation that when compared to normal-adjacent breast tissue, breast tumors exhibit increased heterogeneity [2]. Moreover, our precise identification of differentially methylated CpGs, genes and gene sets allows focused investigation in the future, thereby enabling the identification of effective pharmacologic and therapeutic strategies in the future.

We observed members of the *HOX* gene cluster to be hypermethylated. In line with this, many developmentally related pathways were found to be mildly enriched though not statistically significantly. These findings indicate that development and differentiation-related signaling pathways are characteristic of the HR-deficient, BRCA1-like phenotype. We followed up with this postulate by comparing DNA methylation age - a metric inferred from genome-scale DNA methylation profiles and related to cellular differentiation potential [46] – between BRCA1-like and non-BRCA1-like tumors. In line with our differential methylation and pathway analysis, DNA methylation age was significantly lower in BRCA1-like tumors indicating a more poorly differentiated tumor state. These observations were overall consistent with prior works demonstrating that tumors with *BRCA1/2*-related HR deficiency tend to be poorly differentiated or undifferentiated [57].

Recent studies have shown that *BRCA1*-deficient and *BRCA2*-deficient genomes, despite both having HR loss, may nevertheless differ [15–17]. Therefore, to better understand HR deficiency and chemotherapeutic sensitivity, development and characterization of molecular signatures that more broadly characterize the HR-deficient phenotype may be necessary.

One challenge was identifying HR-deficient, BRCA1-like tumors using a strict probabilistic threshold. Here, we used the cutoff of 0.50, which could be rather conservative. Despite having used a robust cross validation-based machine learning approach, there will be opportunities in the future to potentially improve the performance of our SVM classifier, with better balance among breast cancer subtypes in the training data. We acknowledge the limitation of cell lines in the experimental validation set, and note that future studies would benefit from inclusion of larger human sample sets for validation. Biologically, as seen in the TALORx trial where younger patients (age < 50 years) had improved chemotherapy response [14], we also suspected that confounders such as patient age strata could influence the performance of BRCA1-like classifiers and the molecular characteristics of these tumors. While our Kaplan-Meier analysis showed some evidence that ER/PR-positive, HER2-negative breast cancer with the BRCA1-like phenotype was associated with worse overall survival, our results were not statistically significant in a covariate-adjusted Cox regression model. A possible explanation is the heterogeneity of the treatment regimens among study participants. We therefore anticipate that the application of our SVM BRCA1-like classifier to cohorts with more consistent treatment will have greater clinical value.

## Conclusions

In this work, we applied a copy-number-based classifier to identify breast tumors with the BRCA1-like phenotype. Among breast tumors expressing ER, PR, and/or HER2, we found evidence for previously unknown molecular alterations, including enhanced mutational burden and proliferative capacity, to be associated with the BRCA1-like phenotype. Importantly, we demonstrated that genome-wide DNA methylation profiles differ substantially in HR-deficient, BRCA1-like cancers. The BRCA1-like phenotype may ultimately contribute to increased heterogeneity of molecular alterations in this tumor subset [56], a common characteristic of aggressive but more treatable cancers.

## Additional files

Additional file 1: **Table S1.** SVM BRCA1-like status and BRCA1ness-MLPA profiles in 10 breast cancer cell lines. **Table S2A.** TCGA breast tumors with SVM BRCA1-like status. **Table S2B.** METABRIC breast tumors with SVM BRCA1-like status. **Table S3.** Complete subject characteristics of TCGA and METABRIC breast tumors with SVM BRCA1-like status. **Table S4.** Differentially methylated CpGs in BRCA1-like tumors identified by DMRcate. **Table S5A.** Hypermethylated DMRs (*n* = 108) from DMRcate analysis. **Table 5SB.** Hypomethylated DMRs (*n* = 94) from DMRcate analysis. **Table S6A.** GOBP terms associated with 158 unique genes from 108 hypermethylated DMRs. **Table S6B.** GOBP terms associated with 131 unique genes from 94 hypermethylated DMRs. (XLSX 923 kb)

Additional file 2: **Figure S1.** Details of SVM BRCA1-like classifier. (A) Overview of copy number mapping algorithm for generating the input for training the SVM BRCA1-like classifier. (B) Receiver-operation characteristic curves (ROC) of the classifier applied to training and test set (AUC = 1.00 and 0.75, respectively). (C-D) Correlation of SVM BRCA1-like probability scores with published HR-deficiency metrics (HRD-LOH and LST scores). ***P < 0.001. **Figure S2.** Comparison of Ki-67 (*MKI67*) gene expression as a surrogate marker for cellular proliferation in METABRIC hormone-receptor-positive breast tumors. *P* value indicates statistical significance from a linear model adjusting for age, tumor stage, ER, PR and HER2 positivity. ***P < 0.001. **Figure S3.** Five-year overall survival comparison between BRCA1-like and non-BRCA1-like ER-positive/PR-positive, HER2-negative breast tumors in TCGA and METABRIC (combined). Table inset shows hazards ratio (95% CI) and *P* value from Cox proportional hazards regression adjusting for potential confounders. ***P < 0.001. **Figure S4.** miR124–2 with hypermethylation exhibit reduced gene expression in TCGA BRCA1-like receptor positive tumors. *P* value indicates statistical significance from a linear model adjusting for age, tumor stage, and ER, PR, and HER2 positivity. *P < 0.05. **Figure S5.** Comparison of heterogeneity between the “BRCA1-like methylation cluster” and “non-BRCA1-like methylation cluster” generated by hierarchical clustering of 350 most differential CpGs identified by DMRcate (all FDR <0.05 and |log2∆beta| ≥ 3.50). (A) Heat map showing unsupervised clustering (Euclidean distance, complete linkage) of the 350 DMRcate-identified CpGs. (B) Rank-ordered inter-sample variance in beta-values of the 350 differentially methylated CpGs. Horizontal dotted lines indicate mean inter-sample variance distribution for each group. **Figure S6.** Differential gene expression of DNA methyltransferases (*DNMT1/3A/3B*) in TCGA receptor-positive BRCA1-like breast tumors. A *P* value indicates statistical significance from linear model adjusting for age, tumor stage, and ER, PR, and HER2 positivity. ***P < 0.001. **Figure S7.** Comparison of Somatic Mutational Signature 1 contributed to by genome-wide cytosine-to-thymine (C > T) deamination events in TCGA hormone-receptor-positive breast tumors. A *P* value indicates statistical significance from linear model adjusting for age, tumor stage, and ER, PR, and HER2 positivity. **P < 0.01. (PDF 3058 kb)

## Abbreviations

450 K: Illumina HumanMethylation450 BeadChip
aCGH: Array comparative genomic hybridization
CCLE: Cancer Cell Line Encyclopedia
CpG: Cytosine-phosphate-guanine
DMR: Differentially methylated region
ER: Estrogen receptor
HER2: Human epidermal growth factor receptor 2
HR: Homologous recombination
METABRIC: Molecular Taxonomy of Breast Cancer International Consortium
PR: Progesterone receptor
SVM: Support vector machine
TCGA: The Cancer Genome Atlas
TNBC: Triple negative breast cancer
