# Supplementary material for: Molecular and epigenetic profiles of BRCA1-like hormone-receptor-positive breast tumors identified with development and application of a copy-number-based classifier
Source: Breast Cancer Res. 2019 Jan 25;21:14. doi: 10.1186/s13058-018-1090-z (PMC6347811; doi:10.1186/s13058-018-1090-z)

Supplemental Figure 1. Details of SVM BRCA1-like classifier. (A) Overview of copy number mapping algorithm for generating the input for training the SVM BRCA1-like classifier. **(B)** Receiver-operation characteristic curves (ROC) of the classifier applied to training and test set (AUC=1.00 and 0.75, respectively). **(C-D)** Correlation of SVM BRCA1-like probability scores with published HR-deficiency metrics (HRD-LOH and LST scores). *** $P < 0.001$.

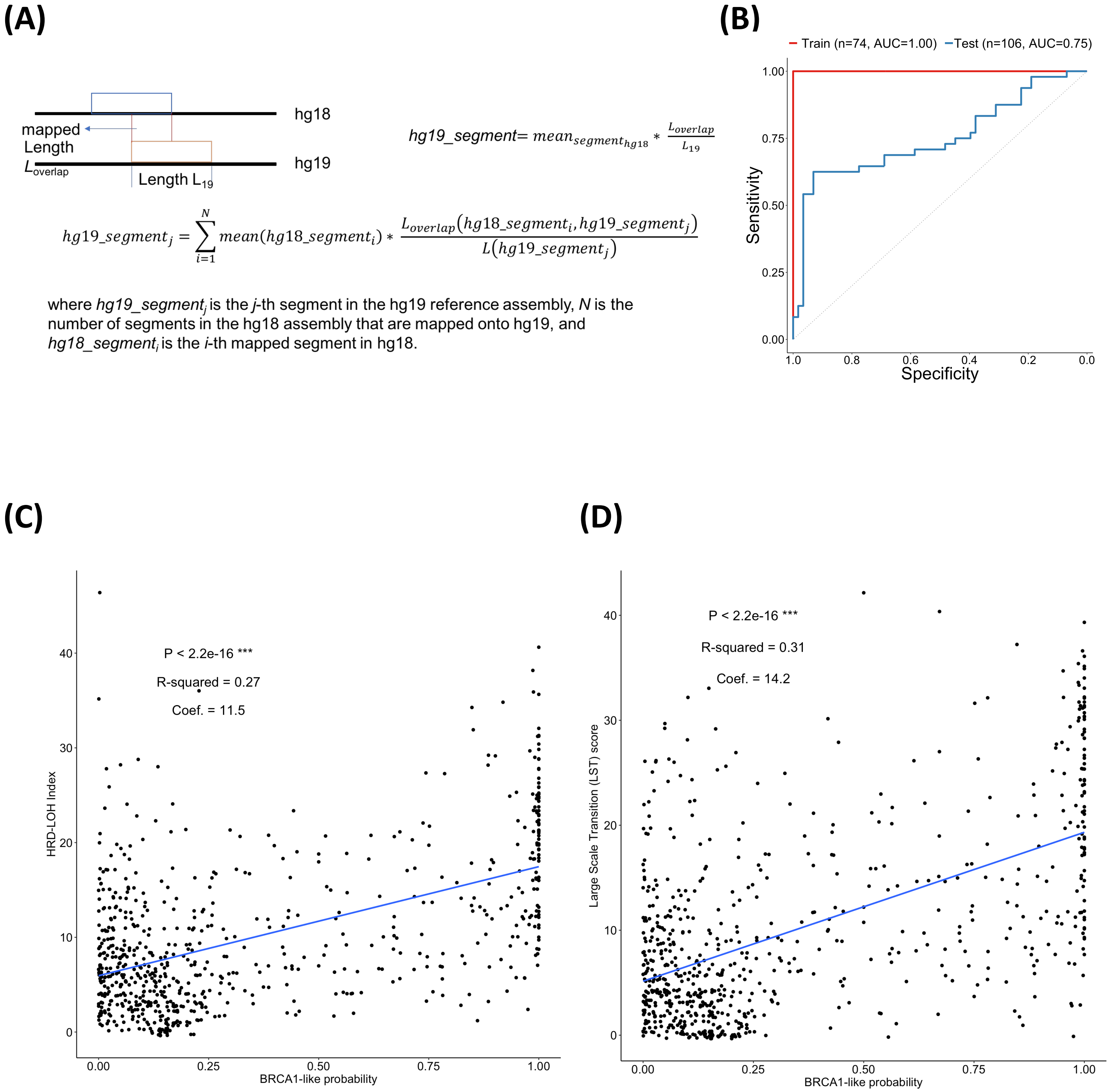

Supplemental Figure 2. Comparison of Ki-67 (*MKI67*) gene expression as a surrogate marker for cellular proliferation in METABRIC hormone receptor-positive breast tumors. *P*-value indicates statistical significance from a linear model adjusting for age, tumor stage, ER, PR and HER2 positivity. *** *P* < 0.001.

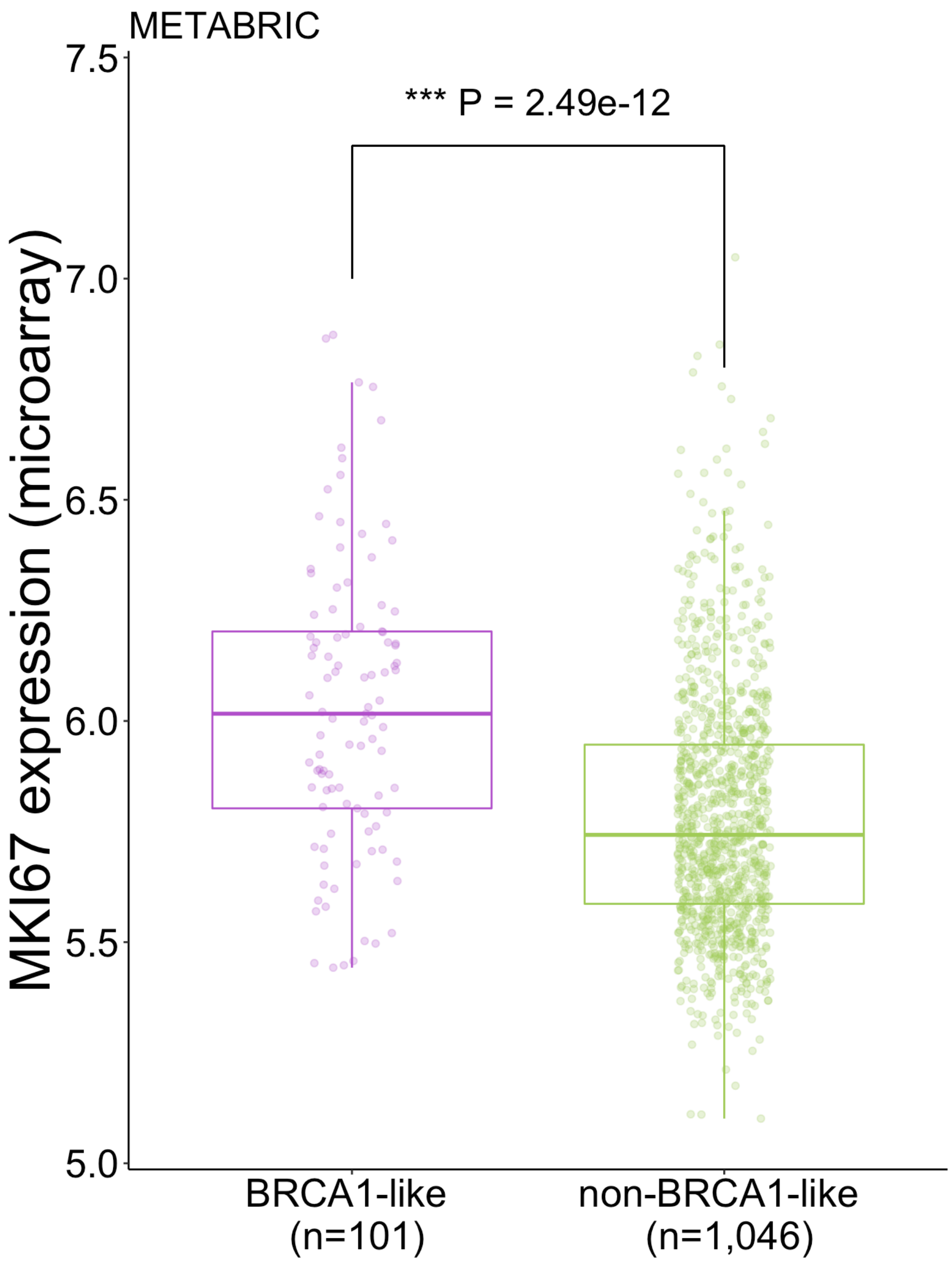

Supplemental Figure 3. Five-year overall survival comparison between BRCA1-like and non-BRCA1-like ER-positive/PR-positive, HER2-negative breast tumors in TCGA and METABRIC (combined). Table inset shows hazards ratio (95% CI) and *P*-value from Cox proportional hazards regression adjusting for potential confounders. ****P* < 0.001.

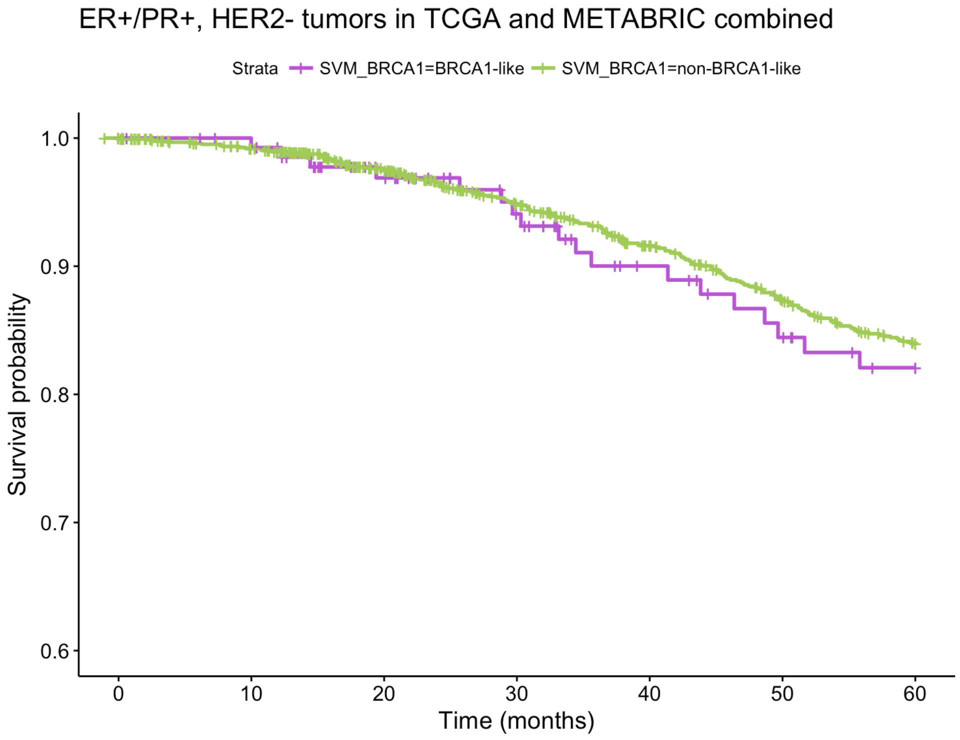

	Combined TCGA and METABRIC (ER+/PR+, HER2-)		
	**n (%)**	**Hazards Ratio (95% CI)**	***P*-value**
**Age** (years)	1,391 (100.0)	1.04 (1.03-1.05)	*** 3.38E-10
**Stage**			
Stage I-II	1,225 (88.1)	1.00 (referent)	
Stage III-IV	166 (11.9)	2.74 (1.89-3.96)	*** 8.95E-08
**SVM BRCA1-like status**			
non-BRCA1-like	1,248 (89.7)	1.00 (referent)	
BRCA1-like	143 (10.3)	1.18 (0.73-1.92)	0.50

Supplemental Figure 4. miR124-2 with hypermethylation exhibit reduced gene expression in TCGA BRCA1-like receptor positive tumors. *P*-value indicates statistical significance from a linear model adjusting for age, tumor stage, ER, PR and HER2 positivity. *$P < 0.05$.

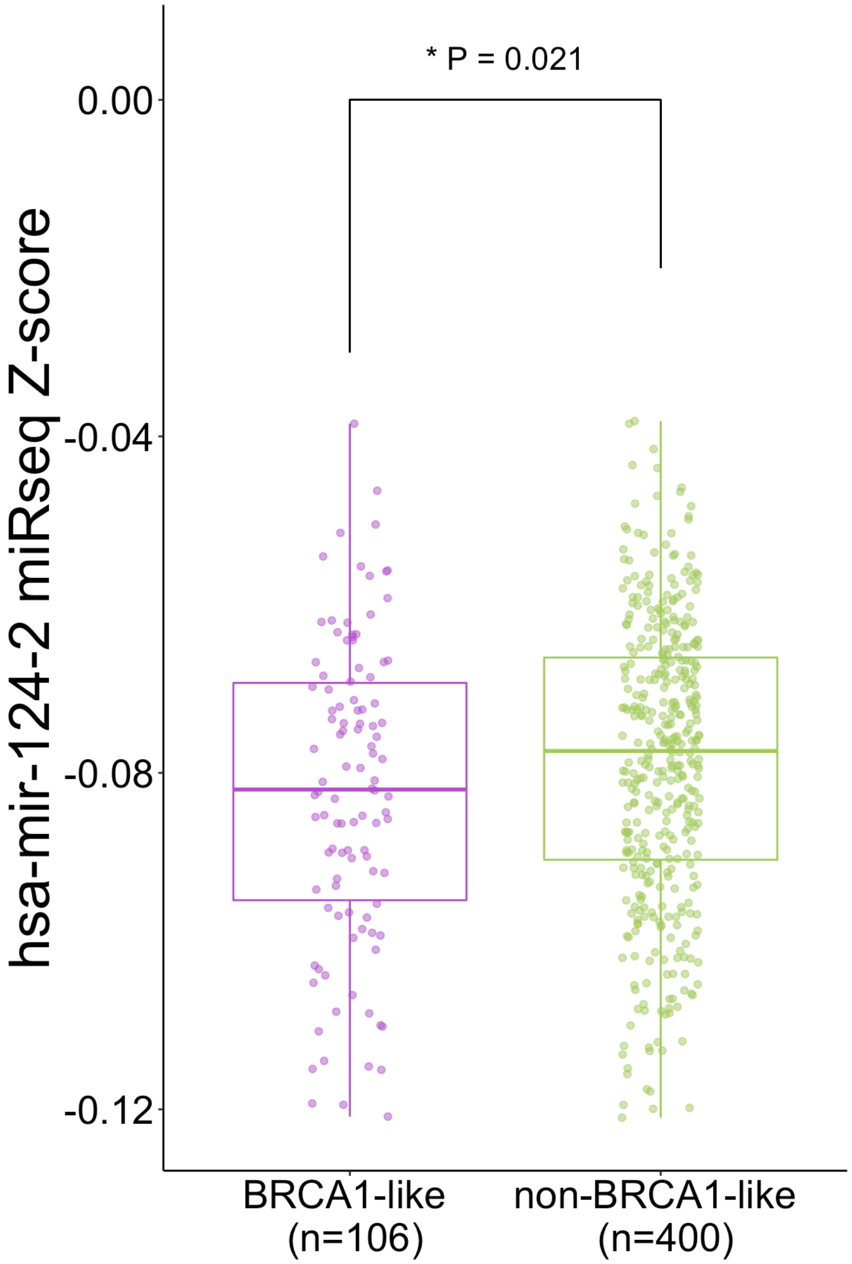

Supplemental Figure 5. Comparison of heterogeneity between the "BRCA1-like methylation cluster" and "non-BRCA1-like methylation cluster" generated by hierarchical clustering of 350 most differential CpGs identified by DMRcate (all FDR < 0.05 and |log2Δbeta|≥3.50). (A) Heat map showing unsupervised clustering (Euclidean distance, complete linkage) of the 350 DMRcate-identified CpGs. **(B)** Rank-ordered inter-sample variance in beta-values of the 350 differentially methylated CpGs. Horizontal dotted lines indicate mean inter-sample variance distribution for each group.

(A)

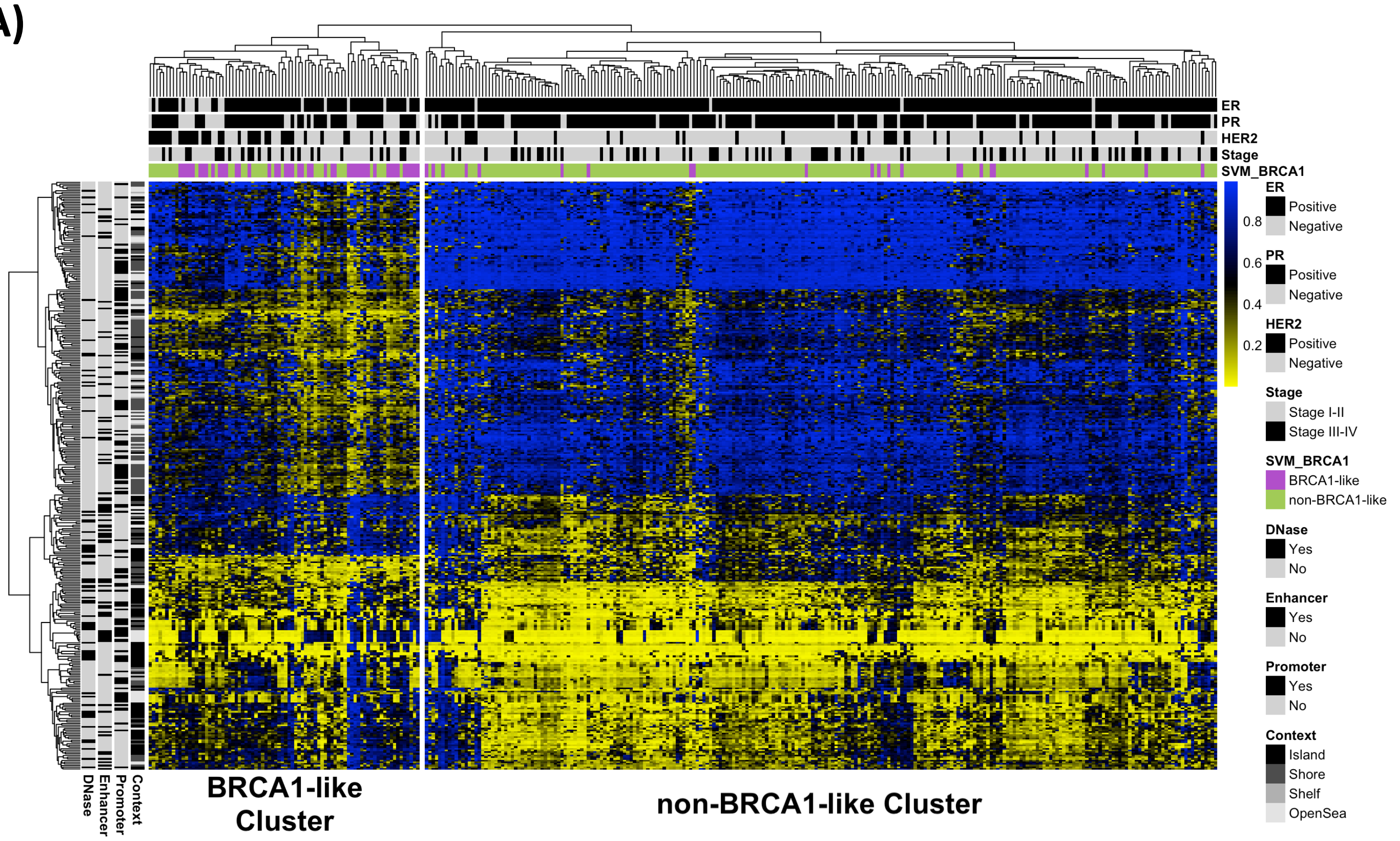

(B)

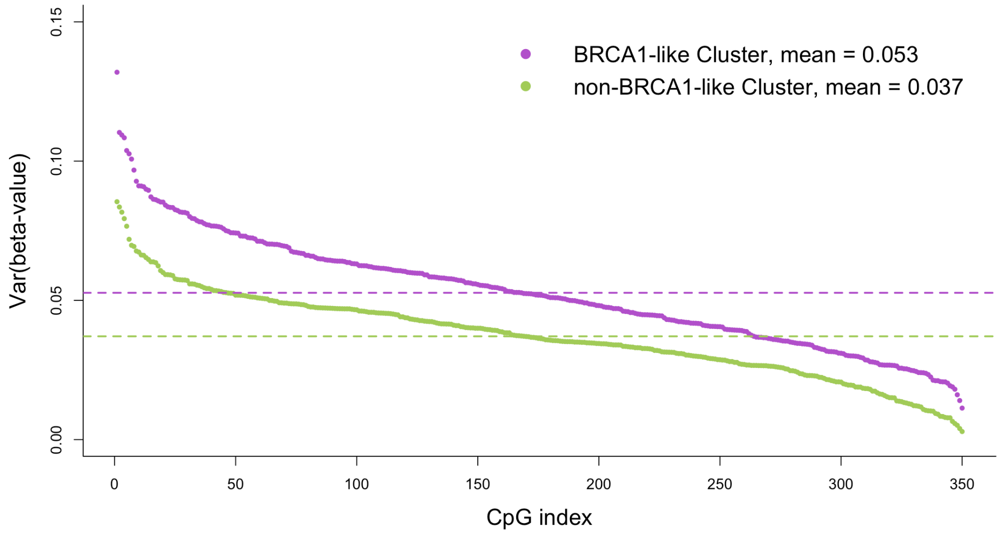

Supplemental Figure 6. Differential gene expression of DNA methyltransferases (*DNMT1/3A/3B*) in TCGA receptor-positive BRCA1-like breast tumors. A *P*-value indicates statistical significance from linear model adjusting for age, tumor stage, ER, PR and HER2 positivity. ***$P < 0.001$.

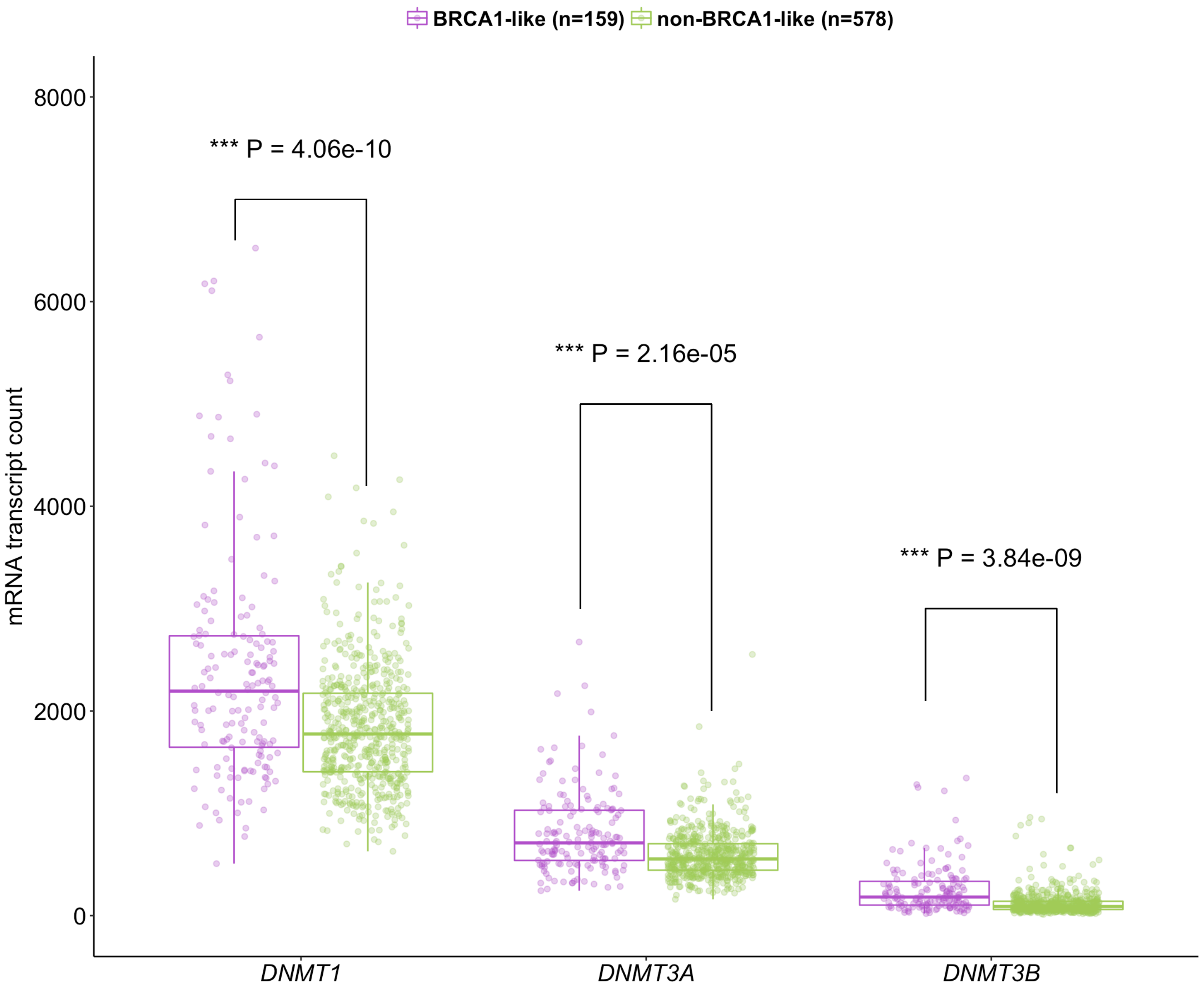

Supplemental Figure 7. Comparison of Somatic Mutational Signature 1 contributed to by genome-wide cytosine-to-thymine (C>T) deamination events in TCGA hormone receptor-positive breast tumors. A *P*-value indicates statistical significance from linear model adjusting for age, tumor stage, ER, PR and HER2 positivity. $^{**}P < 0.01$.

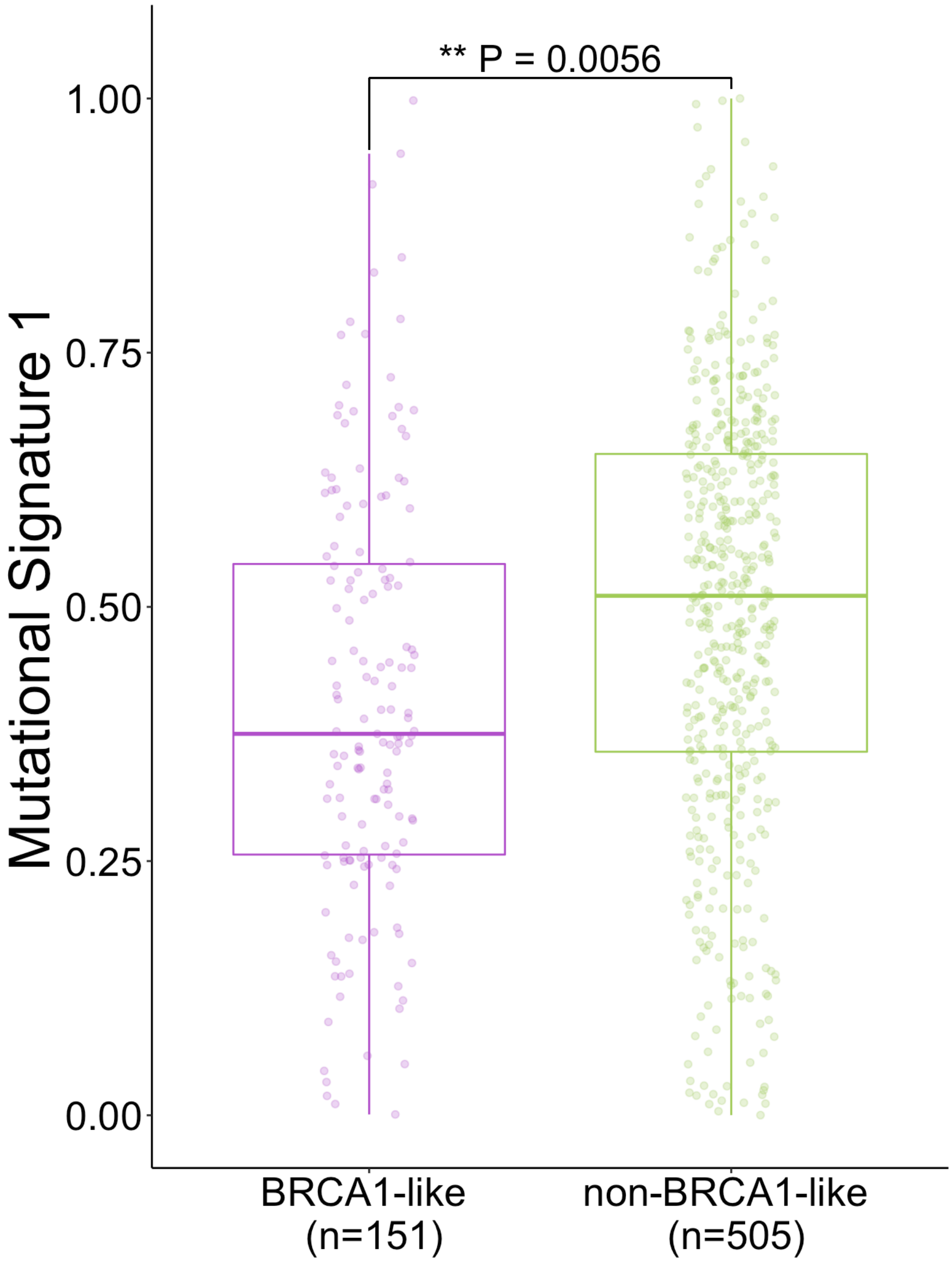

Supplement: Supplementary file 2 — Figure S1. Details of SVM BRCA1-like classifier. (A) Overview of copy number mapping algorithm for generating the input for training the SVM BRCA1-like classifier. (B) Receiver-operation characteristic curves (ROC) of the classifier applied to training and test set (AUC = 1.00 and 0.75, respectively). (C-D) Correlation of SVM BRCA1-like probability scores with published HR-deficiency metrics (HRD-LOH and LST scores). ***P < 0.001. Figure S2. Comparison of Ki-67 (MKI67) gene expression as a surrogate marker for cellular proliferation in METABRIC hormone-receptor-positive breast tumors. P value indicates statistical significance from a linear model adjusting for age, tumor stage, ER, PR and HER2 positivity. ***P < 0.001. Figure S3. Five-year overall survival comparison between BRCA1-like and non-BRCA1-like ER-positive/PR-positive, HER2-negative breast tumors in TCGA and METABRIC (combined). Table inset shows hazards ratio (95% CI) and P value from Cox proportional hazards regression adjusting for potential confounders. ***P < 0.001. Figure S4. miR124–2 with hypermethylation exhibit reduced gene expression in TCGA BRCA1-like receptor positive tumors. P value indicates statistical significance from a linear model adjusting for age, tumor stage, and ER, PR, and HER2 positivity. *P < 0.05. Figure S5. Comparison of heterogeneity between the “BRCA1-like methylation cluster” and “non-BRCA1-like methylation cluster” generated by hierarchical clustering of 350 most differential CpGs identified by DMRcate (all FDR <0.05 and |log2∆beta| ≥ 3.50). (A) Heat map showing unsupervised clustering (Euclidean distance, complete linkage) of the 350 DMRcate-identified CpGs. (B) Rank-ordered inter-sample variance in beta-values of the 350 differentially methylated CpGs. Horizontal dotted lines indicate mean inter-sample variance distribution for each group. Figure S6. Differential gene expression of DNA methyltransferases (DNMT1/3A/3B) in TCGA receptor-positive BRCA1-like breast tumors. A P [file 13058_2018_1090_MOESM2_ESM.pdf]
